# Seasonal Related Multifactorial Control of Pituitary Gonadotropin and Growth Hormone in Female Goldfish: Influences of Neuropeptides and Thyroid Hormone

**DOI:** 10.3389/fendo.2020.00175

**Published:** 2020-04-07

**Authors:** Yifei Ma, Claudia Ladisa, John P. Chang, Hamid R. Habibi

**Affiliations:** ^1^Department of Biological Sciences, University of Calgary, Calgary, AB, Canada; ^2^Department of Biological Sciences, University of Alberta, Edmonton, AB, Canada

**Keywords:** Gonadotropin-inhibitory hormone (GnIH), Gonadotropin-releasing hormone (GnRH), growth, reproduction, thyroid hormone, female, seasonality

## Abstract

Female reproduction is under multifactorial control of brain-pituitary-peripheral origin. The present study provides information on seasonal changes in circulating LH and GH concentrations, as well as transcript levels for a number of genes involved in the regulation of reproduction and growth in female goldfish. We also provide information on the effects of treatments with GnRH and/or GnIH, and their interaction with T3, at three stages of gonadal recrudescence. Maximum basal concentration of LH was observed at late recrudescence (Spring) while no seasonal changes in basal serum GH levels was detected. Serum LH and GH levels were stimulated by GnRH as expected, depending on the season. GnIH stimulated basal GH concentrations in gonadally regressed fish. GnIH inhibitory action on GnRH-induced LH response was observed in late, but not in mid recrudescence. T3 actions on basal and GnRH- or GnIH-induced GH secretion were generally inhibitory, depending on season. Administration of T3 attenuated GnRH-induced LH responses in mid and late stages of gonadal recrudescence, and the presence of GnIH abolished inhibitory actions of T3 in fish at mid recrudescence. Our results also demonstrated seasonal patterns in basal and GnRH- and/or GnIH-induced transcript levels for ERα, ERβI, FSHR, aromatase, TRαI, TRβ, IGF-I, and Vtg in the liver and ovary. However, there were no clear correlations between changes in transcript levels and circulating levels of LH and GH. The results support the hypothesis that GnRH, GnIH, and T3 are contributing factors in complex reciprocal control of reproduction and growth in goldfish.

## Introduction

In most seasonal reproducing oviparous species including fish, reproduction and growth cycles are usually not in-phase with one another because of the significant energy allocation needed to sustain each of these processes. The shift between reproduction and growth phase is associated with changes in the neuroendocrine control by hormones of brain-pituitary-peripheral axis, as well as the accompanying alterations in metabolism. Gonadotropin-releasing hormone (GnRH) is an important neuroendocrine regulator of reproduction because of its ability to stimulate the release and gene expression of pituitary gonadotropins, follicle-stimulating hormone (FSH) and luteinizing hormone (LH). Gonadotropins in turn promote gamete production and hormone production in the ovary and testis. Growth and metabolism are regulated by pituitary growth hormone (GH). GH production and secretion is stimulated by several neurohormones including GnRH (1–4). There are a number of vertebrate GnRH molecular forms and all vertebrates, including mammals, express multiple isoforms of GnRH (5). All vertebrates studied express GnRH2 in different parts of the brain, including the mid brain, and GnRH2 has been suggested to act as a neuromodulator of behavior, food intake, and energy balance rather than as a hypophysiotropic factor (6–8). GnRH3 is only found in teleosts and in species where the predominant vertebrate preoptic hypothalamic GnRH1 form is absent, GnRH3 acts as the main pituitary regulator (5). In goldfish, chicken (c)GnRHII (GnRH2) and salmon (s)GnRH (GnRH3) are the native isoforms (9). There is evidence that both GnRH2 and GnRH3 have hypophysiotropic functions in goldfish and both forms stimulate LH and GH production (2, 4, 5, 10).

While the importance of GnRH in the neuroendocrine regulation of reproduction in vertebrates including fish is well-accepted, there is clear evidence that GnRH is also a key factor in the control of somatotrope activity in fish. In goldfish, GnRH isoforms have been shown to directly stimulate GH release and synthesis from the pituitary (2, 11–16). GnRH binding sites have been observed in somatotrope cells in the pituitary of various fish species including goldfish, cichlids, and pejerry (*Odontesthes bonariensis*) (17–19). GnRH stimulatory actions on GH release has also been demonstrated in other fish species like tilapia (*Oreochromis niloticus*) (20), common carp (*Cyprinus carpio*) (21), and masu salmon (*Oncorhynchus masou*) (22). However, GnRH treatment was found to be without effect on GH production in turbot (*Scophthalmus maximus*) (23), eel (*Anguilla anguilla*) (24), and catfish (*Clarias gariepinus*) (25), indicating that species differences exist even among teleosts.

Multifactorial control of reproduction and growth also involves gonadotropin inhibitory hormone (GnIH). GnIH was first discovered in the Japanese quail (*Coturnix japonica*) as part of the RF-amide family of proteins and GnIH was found to inhibit FSH and LH production and secretion (26). The presence of multiple forms of GnIH and GnIH-related peptide transcripts have been demonstrated in each of a number of species (27). Goldfish have three GnIH genes, although only GnIH-3 (GnIH: SGTGLSATLPQRF-NH2) is expressed in the hypothalamus (28). In mammals and birds species studied, GnIH was found to inhibit gonadotrope functions (29–31). In fish and amphibians, GnIH effects are more complex and appear to be species specific (32). In cinnamon clownfish (*Amphiprion melanopus*), treatment with GnIH inhibited expression of gonadotropin α subunit as well as LHβ, and FSHβ subunits (33). In the cichlid fish (*Cichlasoma dimerus*), GnIH subtypes exert different actions; cdGnIH1 inhibited FSHβ and LH β expression, but cdGnIH2 stimulated FSHβ expression (34). In goldfish, GnIH exerts both stimulatory and inhibitory actions on gonadotropin and GH production, depending on the season (14, 35, 36). Treatment with GnIH inhibited both release and synthesis of LH during early recrudescence but not in late recrudescence goldfish (36). In grass puffer (*Takifugu alboplumbeus*), transcript levels for GnIH, FSH, and LH were found to be higher during spawning period, both *in vitro* and *in vivo* (37). GnIH also increased GH mRNA levels in grass puffer primary pituitary cell cultures (38) and GH release in cichlid *C. dimerus* (34). Similarly, GnIH stimulated GH release in mammals (39, 40). In static incubation of primary cultures of goldfish pituitary cells, GnIH stimulated GH release and mRNA expression in cells from goldfish in late recrudescence; however, GnIH reduced GH mRNA expression in cells obtained from early and mid recrudescence stage fish (14). *In vivo* application of GnIH to goldfish in early, mid, and late stages of recrudescence decreased serum GH concentration but increased pituitary GH mRNA levels (14). Overall, these studies have demonstrated GnIH has both stimulatory and inhibitory effects on LH and GH response that are species specific and seasonally related. To further add to the complexity of GnIH effects, many of these studies utilized mixed sex groups. The complete picture of GnIH regulation of somatotropes and gonadotropes in females remains to be clarified.

In addition to GnRH and GnIH, thyroid hormones (T3 and T4) also play important roles in the control of growth and reproduction. Thyroid hormones are also important factors involved in metamorphosis in amphibians and certain fish species (41–43). Thyroid hormones are known to work in concert with GH to increase growth and metabolism (44–47). Concomitant treatments of T4 and GH in Ames dwarf mice can increase body mass and growth to levels similar to regular non-dwarf mice (48). In contrast, thyroid hormones have both inhibitory and stimulatory effects on somatotrope functions in teleost species. For example, thyroid hormones directly inhibited GH release and synthesis in eel pituitary (49) but *in vivo* T3 treatments increased GH mRNA expression in rainbow trout (*Oncorhynchus mykiss*) (50) and had no effects in goldfish (51). A number of studies have demonstrated actions of thyroid hormone on reproduction in both female and male (52–56). In murrel, *Channa gachua*, and a carp, *Catla catla*, as well as Ranid frogs, GnRH variants increased plasma T4 levels (57, 58), although no changes in T3 levels were observed in the goldfish following GnRH treatment (59). Thyroid hormone levels in goldfish were found to undergo seasonal changes, increasing to maximum levels during the growth phase, and reaching a nadir during spawning (60). While injection with T3 reduced circulating estradiol (E2) level in male, it had no effect in female goldfish (51). T3 treatment increased vitellogenin (Vtg) mRNA levels in the liver of goldfish by increasing expression of estrogen receptor (ER)α mRNA levels at mid stages of gonadal recrudescence (61). In isolated rainbow trout ovarian follicles, T3 treatments enhanced gonadotropin-induced E2 secretion (53). In male fish, thyroid hormones significantly alter spermatogenesis (56). In zebrafish, T3 treatment was shown to stimulate Sertoli cell and spermatogonia type-A proliferation in testis (62).

The aim of the present study is to investigate the hypothesis that GnRH, GnIH, and T3 are players in the multifactorial regulation of growth and reproduction in female goldfish. To this end, the influence of GnRH and/or GnIH injections on serum LH and GH levels, as well as transcript levels of several liver and ovarian genes important for growth and reproduction were monitored at three distinct ovarian recrudescence phases. Transcripts monitored include liver expression of ERs, Vtg, insulin-like growth factor (IGF-I), and thyroid hormone receptors (TRs); and ovarian expression of ERs, FSH receptor (FSHR), and aromatase. The effects of T3 injection on GnRH-elicited and GnIH-induced serum LH and GH responses were also examined to gain insight into the possible influences of thyroid hormones.

## Materials and Methods

### Animals

Common goldfish (*Carassius auratus*) were purchased from Aquatic Imports (Calgary, AB, Canada; goldfish were imported from fish farm exposed to natural daylight and temperature cycles in Pennsylvania, USA). A total of 360 fish (20 fish per treatment group) was used per season representing different gonadal stages: regressed stage (July-August), mid recrudescence (December–January), late recrudescence (March–April). Average body weight of fish in regressed and mid recrudescence stage were 60 g, average weight for late recrudescence fish was 22 g, all fish were at least 1 year old and post pubertal. The difference in fish size was due to the supplier (Aquatic Imports, Calgary, AB, Canada) not able to provide the large number of fish needed for experiments at the time. Fish were housed in a flow-through system with daylight cycles and temperatures adjusted to match conditions in their previous environment to preserve seasonality and allowed to acclimate for 4–7 days prior to use (Table 1). Goldfish were fed once a day to satiation 2 h after lights on, with commercial flake diet (Nutrafin, Baie d'Urfé, QC, Canada). Buffered tricane methanesulfonate solution (MS-222, 160 mg/l, Sigma Aldrich St Louis, Missouri, USA) was used to anesthetize the animals prior to injection treatments, as well as to euthanize the animals after 24 h of treatment. Gonadal recrudescence stages were determined from visual inspection of goldfish ovaries after euthanization. Gonadal regressed stage is characterized by small ovaries and lack of developed follicles (July–August). Ovaries in mid recrudescence contained follicles that continue to increase in size for maximum levels of vitellogenesis (December-January). In late recrudescence, the ovaries are fully mature and have visible oocytes that are ready for ovulation (March–April). Sample of ovaries, liver, and blood from female fish were used for this study and separated based on the above criteria for gonadal recrudescence stage. All animal protocols were approved by the university's animal care committee and in accordance with the Canadian Council on Animal Care's guidelines.

**Table 1:** Temperature and daylight cycles for goldfish housing in the three seasonal ovarian reproductive stages.

**Month**	**Gonadal phase**	**Water temperature (°C)**	**Daylight (h)**
December–January	Mid recrudescence	14	12
March–April	Late recrudescence	16	14
June–August	Regressed	19	14

*Lights were turned on at 9 a.m. every day and turned off at 9 or 11 p.m. These conditions are appropriate for preservation of gonadal stage of fish and similar to those in their natural environment in Pennsylvania (USA)*.

### Injection Treatments

Hormone treatments were given at 0 and 12 h (9 a.m. and 9 p.m.) as intraperitoneal (ip) injections of 100 μL; fish were sacrificed and samples collected at 24 h after the first injection (9 a.m. the following day). The time course of treatments were chosen based on previous studies demonstrating significant effects of GnRH and gonadal steroids at 12 and 24 h following treatments (2, 13, 14, 35, 36, 63–65). GnRH and GnIH treatments were administered using a factorial design (Table 2) and injected with or without the addition of T3 to the hormone mixture. Mixtures of hormones were administered as a single injection (100 μL) for combined hormone treatments. Double injection method was utilized as a previous study has shown GnRH induced a potentiated response when compared to single injection GnRH in stimulating LH release (66). GnRH (sGnRH: Pyr-HWSYGWLPG-NH2) was purchased from Bachem (Torrance, California, USA). Goldfish GnIH (GnIH: SGTGLSATLPQRF-NH2) was made by the University of Calgary Peptide Services (Calgary, AB, Canada). 3,5,3′ tri-iodothyronine (T3) was bought from Sigma Aldrich (Sigma Aldrich St Louis, Missouri, USA) and dissolved in 0.2 M NaOH then serially diluted using Ultrapure water (Sigma Aldrich St Louis, Missouri, USA) to a concentration of 10 ng/ μL to create a stock T3 solution. All hormones were further diluted with phosphate buffered saline (PBS) prior to injection. Sham injections of PBS were used as controls (PBS+PBS; first injection + second injection). Doses of hormones per fish were adjusted based on the dosages per gram of average weight of fish in each season. Doses of hormone injected were chosen based on previous studies in our lab: 100 ng GnRH/g of fish (wet body weight), 50 ng GnIH/g of fish, and 1 ng T3/g of fish (14, 54, 55). At 24 h, samples of blood, liver and gonads were collected and separated by sex. Only samples from female fish were used for the current study. Serum samples were isolated from blood for radioimmunoassays for GH and LH using well-established protocols (12, 67). All samples were stored at −80°C until various assays were performed. Serum FSH was not measured because of the lack of a goldfish FSH radioimmunoassay.

**Table 2:** Factorial design of hormone combination treatments using sGnRH (GnRH) and goldfish GnIH (GnIH).

**Group**	**0 h**	**12 h**
1	PBS	GnRH
2	GnIH	GnIH
3	GnIH	GnRH
4	GnIH	GnIH & GnRH
5	PBS	PBS
6	PBS	GnIH
7	GnRH	GnRH
8	GnIH & GnRH	GnRH
9	GnIH & GnRH	GnIH & GnRH

*Intraperitoneal injections of GnRH (100 ng/g fish, per gram of fish wet weight) and/or GnIH (50 ng/g fish) occurred at 0 h (9 a.m.) and 12 h (9 p.m.), followed by samples collection at 24 h. Phosphate buffered saline (PBS) was used as sham injections, double sham injections (PBS + PBS; first injection + second injection) were used as controls. T3 experimental groups had the addition of T3 at a dose of 1 ng/g of fish in both 0 and 12 h injections*.

### RNA Extraction and QPCR

Total RNA in samples of ovarian and liver tissue were extracted using Trizol Reagent (Invitrogen, Burlington, ON, Canada) in accordance with manufacturer's instructions. Total RNA quantity and purity was determined by Nanodrop spectrophotometer (Thermo Scientific, Waltham, MA, USA). This was followed by DNase digestion (DNase I, Thermo Scientific, Waltham, MA, USA) and cDNA synthesis (High Capacity Multiscribe cDNA kit, Invitrogen, Burlington, ON, Canada).

Liver and ovarian samples were analyzed with real time quantitative (Q)PCR using previously validated primer sets (Table 3; Table S1) (14, 36, 51, 54, 68). GAPDH and β-actin were used as internal controls for ovary and gonads, respectively, based on their stability of expression between treatment groups. All PCR reactions were run in triplicates using SsoFast Eva Green Supermix (BioRad, Mississauga, ON, Canada). The QPCR thermal cycling steps commenced with an initial denaturing step at 95°C for 2 min which was followed by 36 cycle repeats of 95°C for 15 s, 55–60°C appropriate annealing temperature (Table 3) for 15 s, and a final 72°C extension for 1 min. After QPCR amplification, melt curves for each plate were run to ensure only one product was amplified.

**Table 3:** Primers and annealing temperatures used for mRNA transcript quantification in real time QPCR analysis of liver and ovarian tissues.

**Gene**	**Accession numbers**	**Primer**	**Sequence (5′-3′)**	**Annealing temperature (°C)**
β-actin	AB039726	Forward	CCTCCATTGTTGGCAGACC	57
		Reverse	CCTCTCTTGCTTTGAGCCTC	
GAPDH	KT985226.1	Forward	TGATGCTGGTGCCCTGTATGTAGT	57
		Reverse	TGTCCTGGTTGACTCCCATCACAA	
Vtg	DQ641252	Forward	GAAGTGCGCATGGTGGCTTGTATT	55
		Reverse	AGCTGCCATATCAGGAGCAGTGAT	
IGF-I	GU583648.1	Forward	CAGGGGCATTGGTGTGA	57.1
		Reverse	GCAGCGTGTCTACAAGC	
ERα	AY055725.1	Forward	GAGGAAGAGTAGCAGCACTG	55
		Reverse	GGCTGTGTTTCTGTCGTGAG	
ERβI	AF061269.1	Forward	GGCAGGATGAGAACAAGTGG	55
		Reverse	GTAAATCTCGGGTGGCTCTG	
TRαI	AY973629.1	Forward	AGCCTGCCATGCCAGCC	55
		Reverse	CCTCCTGATCCTCGAAGACC	
TRβ	AY973630.1	Forward	GAGGAGCAGCAGAAGACGG	55
		Reverse	GTTGCCTTGGGCGTTTGTGG	
FSHR	HM347775.1	Forward	CGTCCACAATCCTACCTTCG	56
		Reverse	TGAGAAACGGTGATTAGCGG	
Cyp19a1 (aromatase)	AB009336.1	Forward	TTGTGCGGGTTTGGATCAATGGTG	58
		Reverse	TTCCGATACACTGCAGACCCAGTT	

*All primers were designed and validated using Primer3 online software (Whitehead Institute of Biomedical Research, Cambridge, MA, USA)*.

### Statistical Analysis

A maximum of *n* = 12 randomly selected serum samples were processed for RIAs and a maximum of *n* = 9 samples were used for QPCR. Basal circulating levels of GH and LH in the reproductive seasons were measured in the appropriate control groups, reported as actual concentrations (means ± SEM, ng/ml), and analyzed by one-way ANOVA followed by with Tukey's *post-hoc* honestly significant (HSD) multiple comparison tests. In particular to examine the influence of ip injected T3, hormone levels of experimental treatment groups were further normalized to the averaged values of the control group having the highest hormone concentration among the three seasonal reproductive stages, reported as a percentage value, and analyzed with two-way ANOVA followed by Bonferroni's multiple comparisons tests.

Basal QPCR results were normalized to the appropriate housekeeping gene (β-actin or GAPDH) using the using the ΔΔCq method. Housekeeping genes were chosen based on stability of expression between treatment and control groups, and lowest variation (SD) in Ct values. Based on this criteria GAPDH was chosen for the ovaries and β-actin for the liver. QPCR data of experimental groups were further normalized with respect to the averaged value of the control group with highest basal transcript levels among the three reproductive stages examined. All QPCR results were analyzed by the Kruskal-Wallis test followed by Dunn's multiple comparison test. Kruskal-Wallis test was chosen because some of the data sets did not follow normal distribution. Prism 7 was used for these statistical tests (GraphPad Software Inc., La Jolla, CA, USA). Differences are considered significant when *P* < 0.05.

## Results

### Basal Levels of Serum LH and GH

**Figure 1:**
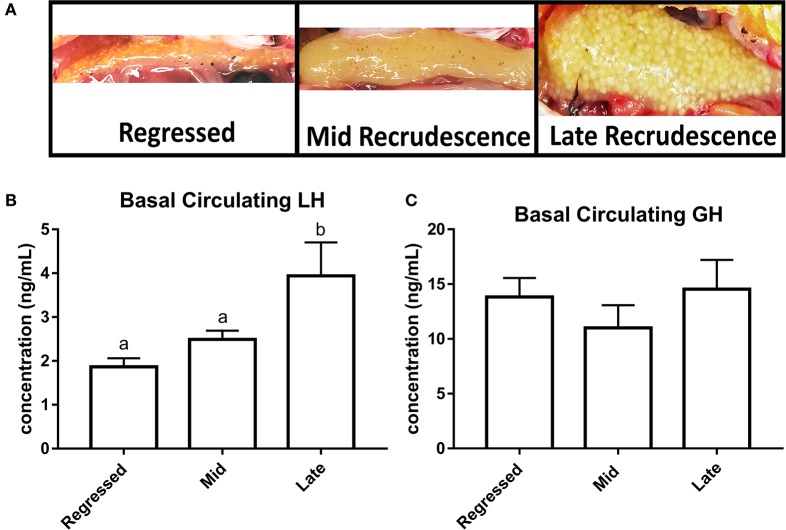
Photographs of ovaries representing different gonadal stages **(A)**. Circulating basal serum levels of LH **(B)** and GH **(C)** in female goldfish at three seasonal reproductive stages (mean ± SEM, *n* = 4–12). Basal hormone levels were taken from control groups (PBS + PBS). Groups with significant differences between one another do not share the same letter of the alphabet (ANOVA followed by Tukey's multiple comparisons test, *p* < 0.05). Where ANOVA did not reveal the presence of significant differences between any of the groups within the whole data set, no identifier is included.

Fish ovaries were visually inspected for gonadal recrudescence status of the goldfish (Figure 1A). The gonadal regressed season starts following ovulation when post-ovulatory ovaries are characterized by their small size and the absence of developed follicles (July–August). Early recrudescence begins when follicular cells multiply and increase in size for higher steroidogenic activity needed for vitellogenin production (September-October). Mid recrudescence ovaries are characterized by follicles that continue to increase in size and this season corresponds to a period of maximum levels of vitellogenesis for yolk production (December–January). Fully matured ovaries with visible oocytes ready for ovulation are present during late recrudescence (March–April). We investigated female goldfish during three stages of recrudescence: regressed, mid, and late recrudescence. Significantly higher levels of LH were observed in serum of fish during late recrudescence compared to regressed and mid recrudescent stages (Figure 1B). Basal circulating GH levels did not significantly change between seasons (Figure 1C).

### Control of Serum LH and GH Levels by GnRH

**Figure 2:**
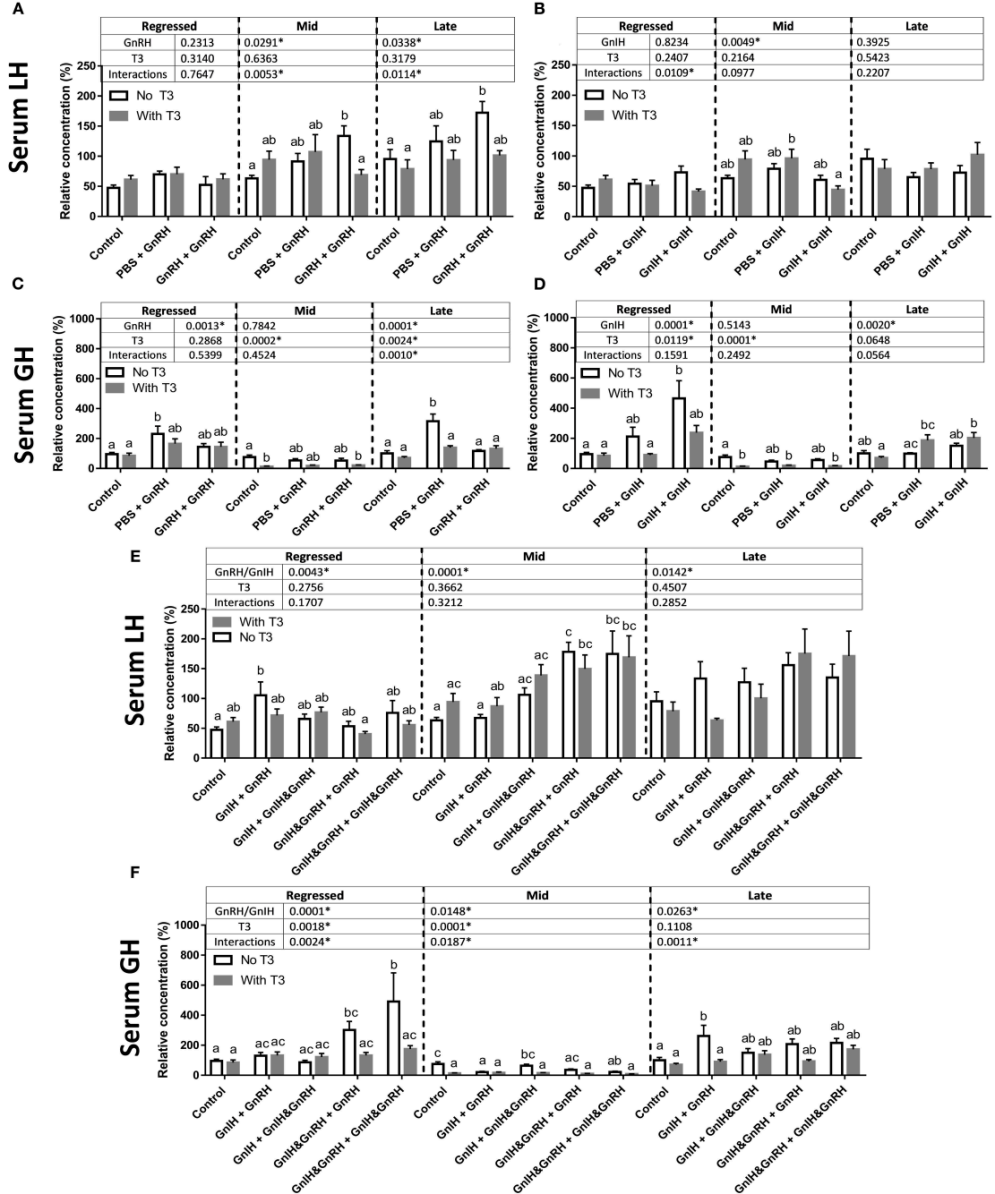
Serum LH level changes in response to GnRH **(A)**, GnIH **(B)**, GnRH and GnIH **(E)** treatments. Serum GH changes in response to GnRH **(C)**, GnIH **(D)**, GnRH and GnIH **(F)** treatments. GnRH- and/or GnIH-induced changes in serum levels in female goldfish in the absence (open bar) and in the presence of T3 (gray bar) at three seasonal reproductive stages (mean ± SEM; regressed *n* = 4–12, mid recrudescence *n* = 4–12, and late recrudescence *n* = 4–9). Treatments are denoted as 0 + 12 h injection. Hormone values are presented as a percentage of the averaged concentrations of to control group (PBS + PBS injected) in late recrudescence. Controls of each respective season were placed at the start of each set of hormone treatments for visual comparison. Within each season, groups identified by different letters are significantly different from one another (two-way ANOVA followed by Bonferroni's multiple comparison test, *p* < 0.05). Vertical dashed line indicates groups within a seasonal gonadal stage that were statistically compared for significance. Groups were not tested for significance between seasons. In seasons where significant differences between groups are absent, identifiers are not included. *P*-values from two-way ANOVA are presented in tables above bars, and asterisks in tables indicate where factors (two factors analyzed in two-way ANOVA: GnRH and/or GnIH treatment, and the addition of T3) had significant effects, *p* < 0.05.

The effects of the pre-optic hypothalamic goldfish GnRH form (sGnRH) (69), applied either as a single or double ip injection, were examined in the present study. Previous studies have shown that two ip injections of GnRH applied 12-h apart were generally more effective than a single GnRH injection in elevating circulating LH levels in goldfish (66), and thus both single and double injection protocols were used in the present study. Two-way ANOVA revealed the presence of GnRH effects on serum LH and GH levels among some of the reproductive stages examined (Figure 2A). Specifically, double injection with GnRH (GnRH + GnRH) did not affect serum LH levels in regressed fish but significantly elevated serum LH concentrations in fish at mid and late recrudescence by ~70% relative to controls (PBS + PBS; Figure 2A). Increases in serum GH levels following single GnRH injection treatment (PBS + GnRH) were observed in regressed phase and late recrudescence as expected, and no significant changes were observed in mid recrudescence (Figure 2C).

### GnIH Effects on Basal and GnRH-Induced Serum LH and GH Levels

Treatment of GnIH was administered following a similar protocol as GnRH alone and combined with GnRH. Although two-way ANOVA suggested the presence of an overall GnIH alone treatment effects in mid recrudescence, treatment with GnIH alone did not significantly alter basal serum LH levels relative to controls during regressed, mid, or late gonadal recrudescence stages (Figure 2B). The combination of GnIH + GnRH treatment resulted in significant elevations in serum LH levels in sexually regressed fish when single injection of GnRH alone had no effects (Figures 2A,E). On the other hand treatment of GnIH tended to blunt GnRH-stimulated LH responses in late stages of recrudescence but did not block GnRH-induced responses in mid stage of recrudescence (Figure 2E). These observations are consistent with the presence of overall combination GnIH and GnRH treatment effects on serum LH at all three ovarian recrudescence stage as revealed by the two-way ANOVA.

Double injection of GnIH exerted significant stimulatory actions on circulating GH levels in fish at regressed stage but not at mid recrudescence (Figure 2D); these observations were consistent with the presence of an overall GnIH influence on serum GH levels at regressed but not late recrudescence. GnIH alone treatments at late stages of recrudescence were also without effect on serum GH levels relative to controls, although an overall influence of GnIH was revealed by two-way ANOVA at this reproductive stage (Figure 2D). Significant effects on combination GnRH and GnIH treatments on serum GH levels were indicated by two-way ANOVA for all three reproductive seasons examined (Figure 2F). GnRH co-injection did not alter GnIH-induced GH responses in the regressed fish (Figure 2F). In mid stages of recrudescence, GnIH + GnRH and GnIH & GnRH + GnIH & GnRH treatments exerted inhibitory effects on serum GH levels but other combination treatments had no effects (Figure 2F). In late stages of recrudescence, GnIH + GnRH treatment increased serum GH to levels similar to those induced by a single GnRH injection while other combination treatments of GnIH and GnRH generally did not alter serum GH levels (Figure 2F).

### Effects of T3 on Basal, and GnRH- and/or GnIH-Induced Changes in LH and GH Levels

Two-way ANOVA identified significant interactions of T3 with GnRH at mid and late recrudescence, as well as interactions with GnIH at regressed state, but no significant overall effects of T3 alone on serum LH nor significant T3 interactions with combination GnRH and GnIH treatments (Figures 2A,B,E). Accordingly, treatment of T3 had no effects on basal LH release during any season but lowered the LH responses to double GnRH injections during mid and late stages of recrudescence (Figure 2A). Combination treatments with T3 and GnIH did not influence circulating LH levels (Figure 2B). T3 treatments with GnRH in the presence of GnIH did not alter GnRH-induced serum LH increases in mid recrudescent fish (Figure 2E). No significant effects were observed in these triple combination hormone treatment groups during regressed and late stage of gonadal recrudescence relative to controls (Figure 2E). Interestingly, the GnIH + GnRH-induced LH response was reduced by T3 injection to a level not different from controls in sexually regressed fish (Figure 2E).

The presence of significant overall T3 effects on serum GH were revealed at all reproductive seasons by two-way ANOVA, and in particular, interactions of T3 were identified with GnRH at late recrudescence, and with combination GnRH and GnIH treatments at all three ovarian stages (Figures 2C,D,F). T3 treatment significantly lowered basal serum GH levels during mid stages of recrudescence (Figure 2D) and reduced GnIH-induced GH response in regressed gonadal stage to levels not different to controls (Figure 2F). T3 also inhibited GnRH-induced GH release in late stages of recrudescence but not in regressed gonadal stage (Figure 2C). In addition, T3 reduced the GH responses to combination GnIH and GnRH treatments in regressed and late recrudescence stages (regressed: GnIH & GnRH + GnRH and GnIH & GnRH + GnIH & GnRH; late recrudescence: GnIH+GnRH; Figure 2F).

### Transcripts in Liver and Ovaries

**Figure 3:**
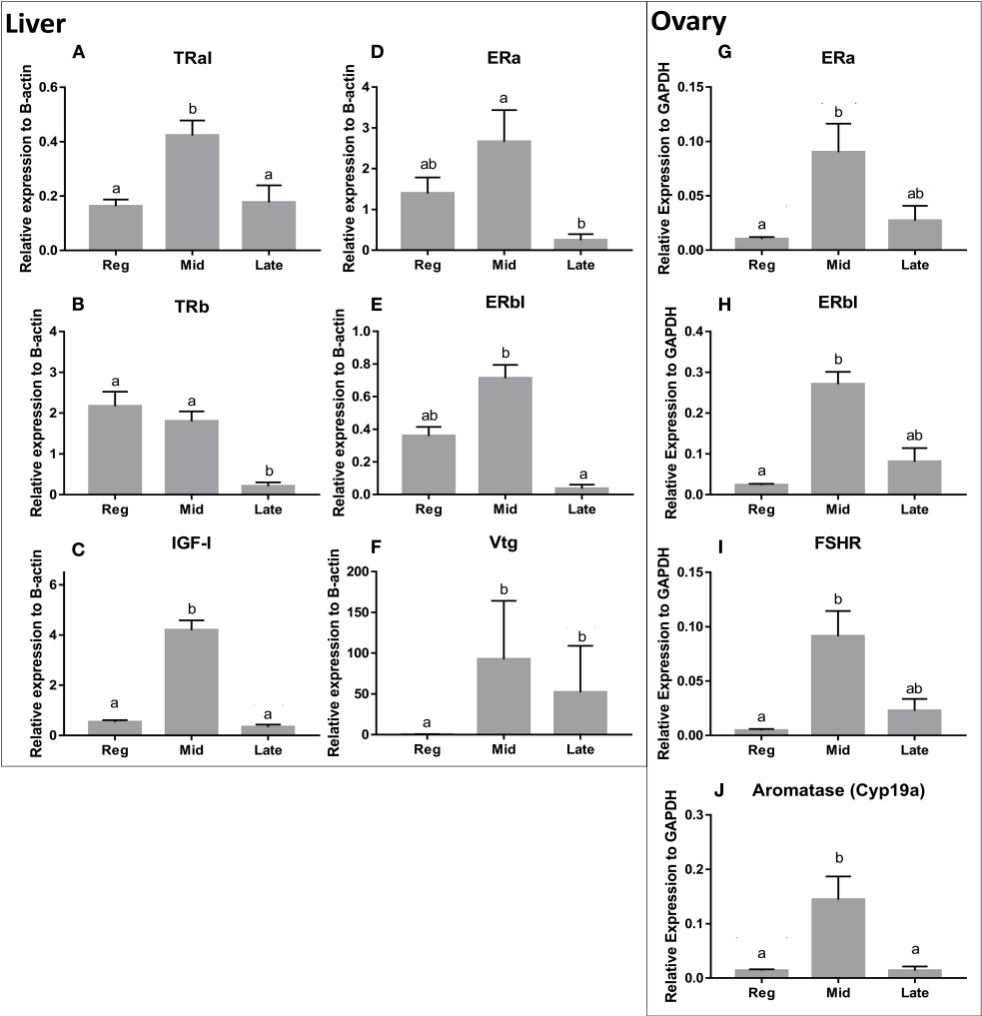
Basal transcript levels of TRαI **(A)**, TRβ **(B)**, IGF-I **(C)**, ERα **(D)**, ERβ1 **(E)**, and vitellogenin **(F)** in the liver, and ERα **(G)**, ERβ1 **(H)**, FSHR **(I)**, and aromatase Cyp19a **(J)** in the ovaries of female goldfish in three stages of the reproductive season (regressed, mid, and late recrudescent). Expression was measured using QPCR and normalized against β-actin (liver) or GAPDH (gonads). Values are mean ± SEM (*n* = 4–9). Groups identified by different letters are significantly different from one another (Kruskal-Wallis test followed by Dunn's multiple comparisons test, *p* < 0.05).

Transcript levels for several genes important in reproduction and growth were measured with QPCR in liver and ovaries. The results for all transcript levels in liver and ovaries demonstrate clear seasonal pattern (Figure 3).

#### ERα

**Figure 4:**
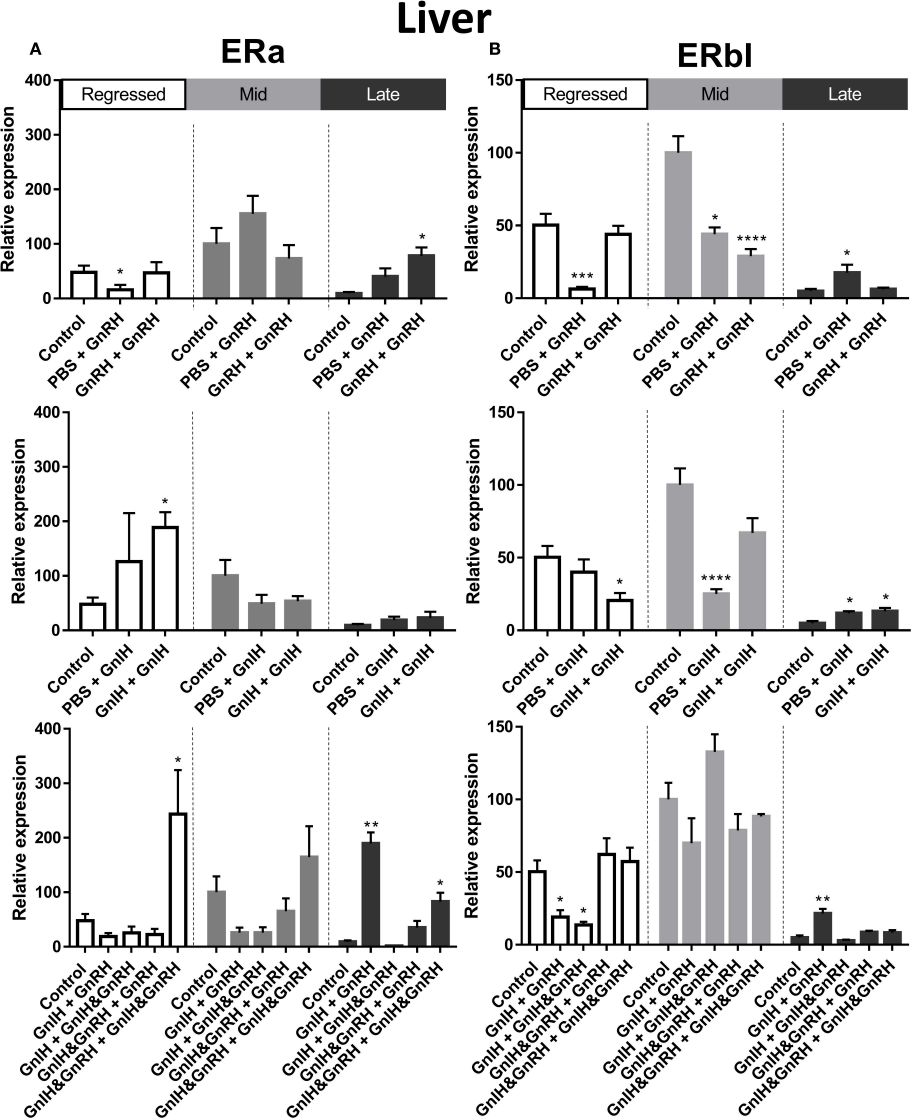
Effects of GnRH and/or GnRH treatments on estrogen receptors ERα **(A)** and ERβ1 **(B)** mRNA levels in the liver of female goldfish at three seasonal reproductive stages (regressed, *n* = 4–9; mid recrudescence, *n* = 4–9; and late recrudescence *n* = 4–9). Treatments are denoted as 0 + 12 h injection. Levels of mRNA expression were detected using QPCR and normalized relative to β-actin. Results presented (mean ± SEM) are expressed as a percentage of the averaged relative expression levels in controls (PBS + PBS) at mid recrudescence. Each seasonal control group was placed in front of each set of hormone treatments for visual comparison. Asterisks indicate significance differences from controls (Kruskal-Wallis test followed by Dunn's multiple comparisons test, *p < 0.05, **p < 0.01, ***p < 0.001, ****p < 0.0001).

**Figure 5:**
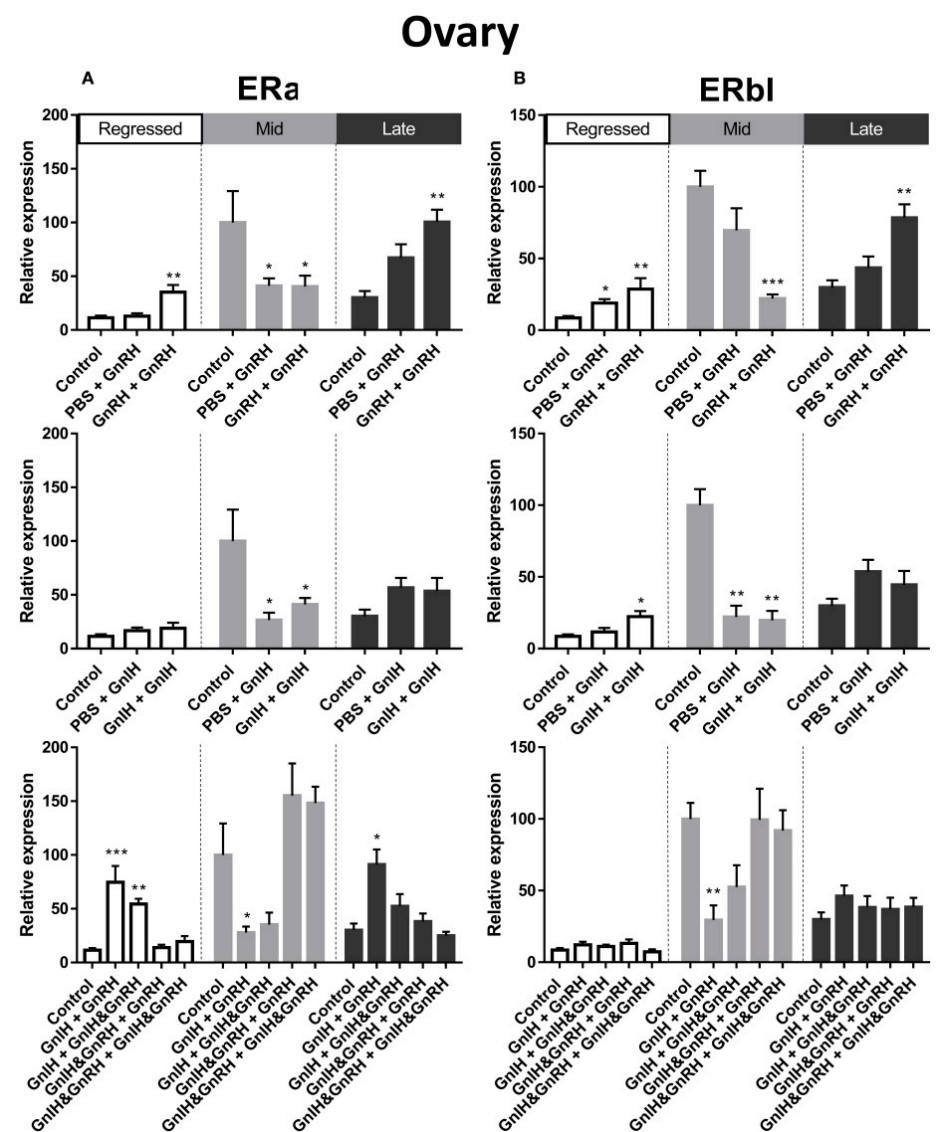
Effects of GnRH and/or GnIH on estrogen receptors ERα **(A)** and ERβ1 **(B)** mRNA levels in the ovary of female goldfish at three seasonal reproductive stages (regressed, *n* = 4–9; mid recrudescence, *n* = 4–9; and late recrudescence *n* = 4–9). Treatments are denoted as 0 + 12 h injection. Levels of mRNA expression were detected using QPCR and normalized relative to GAPDH. Results presented (mean ± SEM) are expressed as a percentage of the averaged relative expression levels in control (PBS + PBS) group at mid recrudescence. Each seasonal control group was placed in front of each set of hormone treatments for visual comparison. Asterisks indicate significance differences from controls (Kruskal-Wallis test followed by Dunn multiple comparisons test, *p < 0.05, **p < 0.01, ***p < 0.001).

ERα mRNA levels in the liver were significantly higher in mid recrudescent fish compared to fish in late recrudescence (Figure 3D). Similar to the changes in liver, ERα mRNA levels in the ovaries were significantly higher in mid stages of recrudescence but were lowest in regressed state fish (Figure 3G). Except for double GnRH injection treatment, changes in ERα mRNA levels in the liver did not correlate well with treatment-induced serum LH levels. GnRH + GnRH. Double GnRH injection treatment elevated ERα transcript levels in liver during late stages of gonadal recrudescence whereas single GnRH injection reduced the level of this transcript at regressed state (Figure 4A). In contrast, elevations in liver ERα mRNA levels were observed following double GnIH injections in regressed gonadal stage, GnIH & GnRH + GnIH & GnRH treatments in regressed and late recrudescence stages, as well as GnIH+GnRH treatments in late recrudescence (Figure 4A). Double GnRH injection treatment increased ERα transcript levels in ovaries during late stages of recrudescence and regressed gonadal stage (Figure 5A); these changes corresponded to the GnRH-induced increases in serum LH concentrations. However, GnIH treatments reduced ovary ERα mRNA levels during mid stages of recrudescence (Figure 5A). Treatment with GnIH+GnRH or GnIH+GnIH & GnRH increased levels of ERα mRNA level in ovaries during regressed gonadal stage (Figure 5A). GnIH+GnRH treatment reduced the levels of ERα mRNA in ovaries at mid recrudescence, but increased their ovarian expression during late recrudescence (Figure 5A).

#### ERβI

There was a clear seasonal pattern in expression of ERβI mRNA in liver and ovaries of female goldfish with maximum levels seen in mid recrudescence, and lowest levels seen in liver of late recrudescence fish and ovaries of regressed stage fish (Figures 3E,H). ERβI mRNA levels in the liver of females showed no correlation with responses in LH concentrations elicited by hormone treatments. Single GnRH injection treatments reduced liver ERβI mRNA levels in sexually regressed fish, but increased these transcript levels in late recrudescence (Figure 4B). All GnRH alone treatments in mid recrudescence lowered ERβI mRNA levels in liver (Figure 4B). Double GnIH injection treatment lowered liver ERβI mRNA levels during regressed and mid recrudescence stages and single GnIH injection similarly exerted inhibitory influences in mid recrudescence (Figure 4B). On the other hand, GnIH treatments increased ERβI mRNA levels in liver during late recrudescence (Figure 4B). Combined treatments with GnIH + GnRH and GnIH + GnIH & GnRH reduced ERβI mRNA levels in liver during regressed stage (Figure 4B). GnIH+GnRH combination treatment elevated ERβI mRNA levels in liver during late recrudescence stage (Figure 4B). In ovaries, ERβI mRNA levels partially correlated with changes in serum LH concentrations in late stages of gonadal recrudescence (Figure 5B). Single and double GnRH injection treatments increased ERβI mRNA levels in ovaries in regressed stage; double GnRH injection treatments also increased these levels in late recrudescence stage, but reduced these levels in mid recrudescence (Figure 5B). Double GnIH injection treatments increased ovarian ERβI mRNA levels in regressed phase fish, but both double and single GnIH injection treatments decreased ERβI mRNA levels in ovaries of fish at mid recrudescence (Figure 5B). GnIH+GnRH treatment reduced ERβI mRNA levels in ovary during mid recrudescence, but no effects were seen in combination GnIH and GnRH treatment groups in other seasons (Figure 5B).

#### Vtg

**Figure 6:**
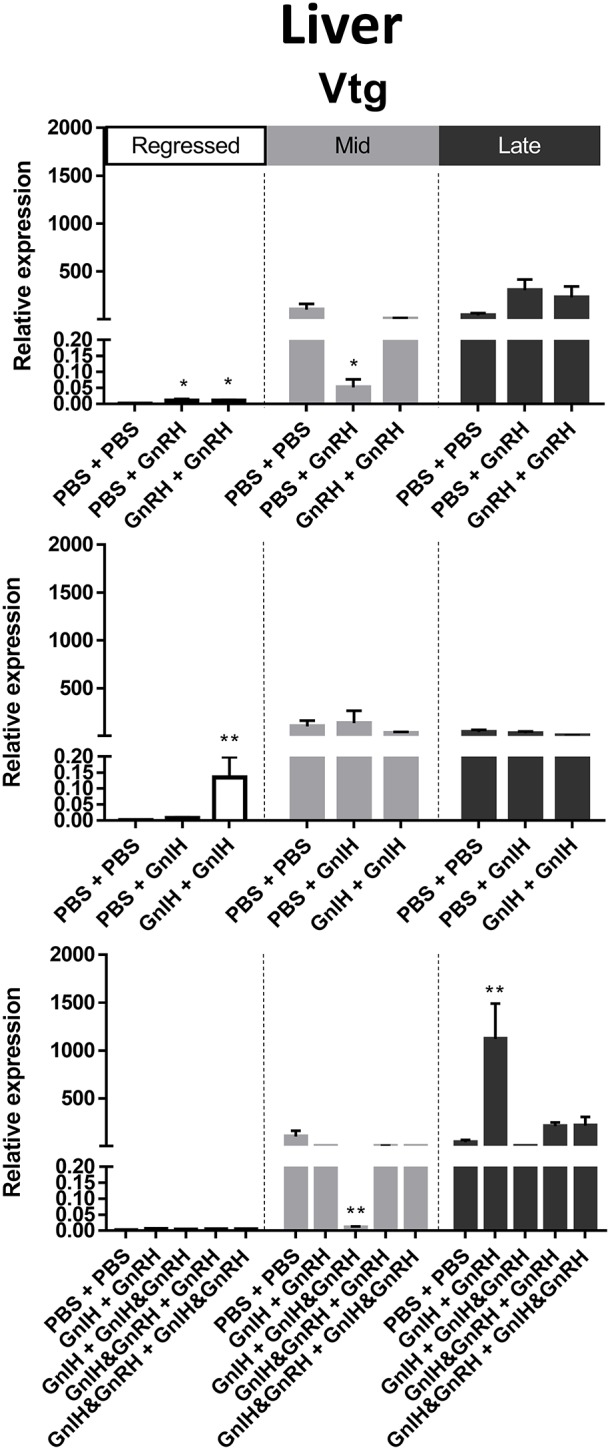
Effects of GnRH and/or GnIH on Vtg mRNA levels in the liver of female goldfish at three seasonal reproductive stages (regressed, *n* = 4–9; mid recrudescence, *n* = 4–9; and late recrudescence *n* = 4–9). Treatments are denoted as 0 + 12 h injection. Levels of mRNA expression were detected using QPCR and normalized relative to β-actin. Results presented (mean ± SEM) are expressed as a percentage of the averaged relative expression levels in control (PBS + PBS) group at mid recrudescence. Each seasonal control group was placed in front of each set of hormone treatments for visual comparison. Asterisks indicate significance differences from controls (Kruskal-Wallis test followed by Dunn's multiple comparisons test, *p < 0.05, **p < 0.01).

Liver Vtg mRNA levels were significantly higher in mid and late recrudescence compared to regressed stage (Figure 3F). There was a correlation between basal Vtg mRNA levels and changes in basal circulating LH levels in late recrudescence. Both single and double GnRH injection treatments increased Vtg mRNA levels in regressed stage females but single GnRH injection and GnIH + GnIH & GnRH treatments lowered these levels in mid recrudescence stage (Figure 6). Treatments with GnRH did not elicit any significant changes in Vtg mRNA levels in the liver of females during late recrudescence (Figure 6). In regressed gonadal stage, double GnIH injection elevated Vtg mRNA levels in liver while GnIH + GnRH treatment was similarly stimulatory at late recrudescence (Figure 6). Other concomitant treatments with GnRH and GnIH were without effect on the liver Vtg mRNA level in the three seasons (Figure 6).

#### IGF-I

**Figure 7:**
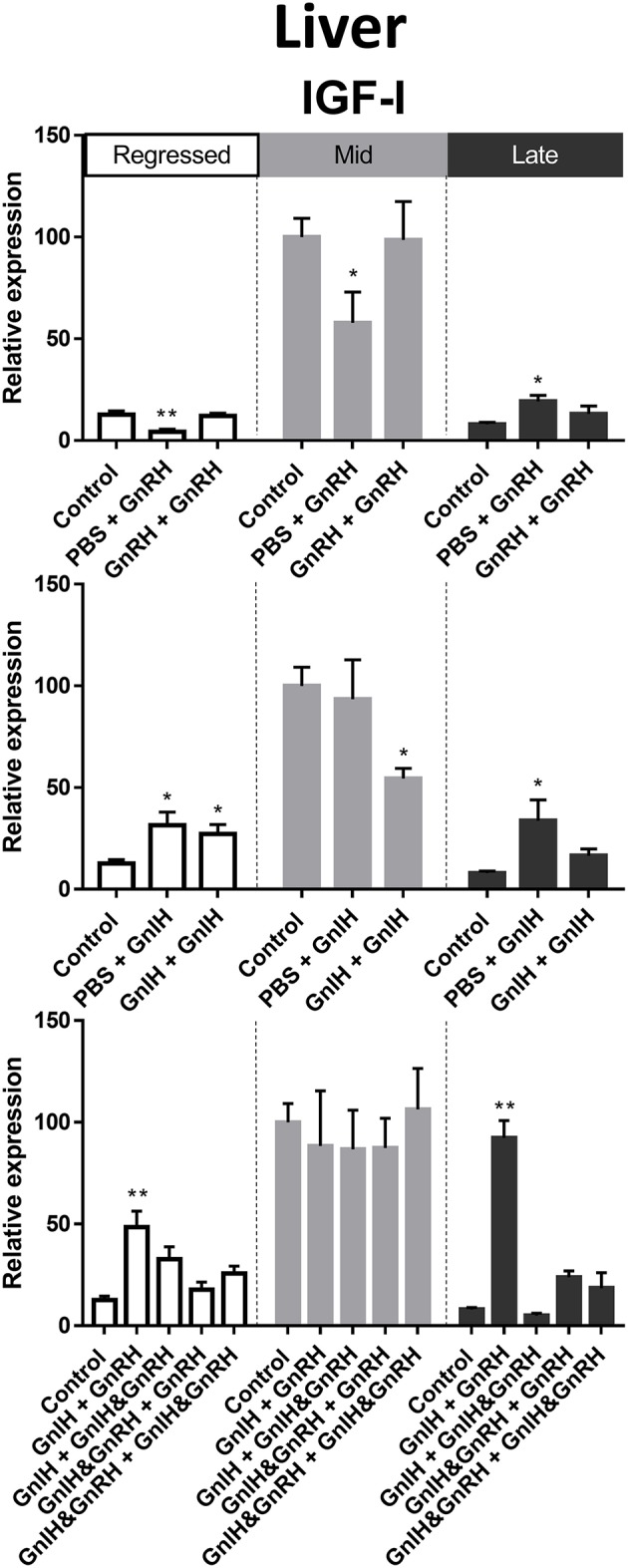
Effects of GnRH and/or GnIH on IGF-I mRNA levels in the liver of female goldfish at three seasonal reproductive stages (regressed, *n* = 4–9; mid recrudescence, *n* = 4–9; and late recrudescence *n* = 4–9). Treatments are denoted as 0 + 12 h injection. Levels of mRNA expression were detected using QPCR and normalized relative to β-actin. Results presented (mean ± SEM) a

In female liver, IGF-I mRNA levels were significantly higher in mid recrudescence (Figure 3C). A partial correlation was observed between changes in IGF-I mRNA level and serum GH concentration in response to hormone treatments during late stages of gonadal recrudescence. Single GnRH injection treatment reduced IGF-I mRNA levels in liver during regressed and mid recrudescence gonadal stage, but elevated IGF-I mRNA levels during late recrudescence (Figure 7). Single and double injection of GnIH increased IGF-I mRNA levels in liver at regressed stage, and single injection similarly increased mRNA levels in late recrudescent; on the other hand, treatment with double GnIH injection decreased IGF-I mRNA levels in liver during mid recrudescence (Figure 7). No significant effects were observed in combination treatment during mid recrudescence stage (Figure 7), but GnIH + GnRH treatment increased IGF-I mRNA levels in liver during regressed and late recrudescence stages (Figure 7).

re expressed as a percentage of the averaged relative expression levels in control (PBS + PBS) group at mid recrudescence. Each seasonal control group was placed in front of each set of hormone treatments for visual comparison. Asterisks indicate significance differences from controls (Kruskal-Wallis test followed by Dunn multiple comparisons test, *p < 0.05, **p < 0.01).

#### TRs

**Figure 8:**
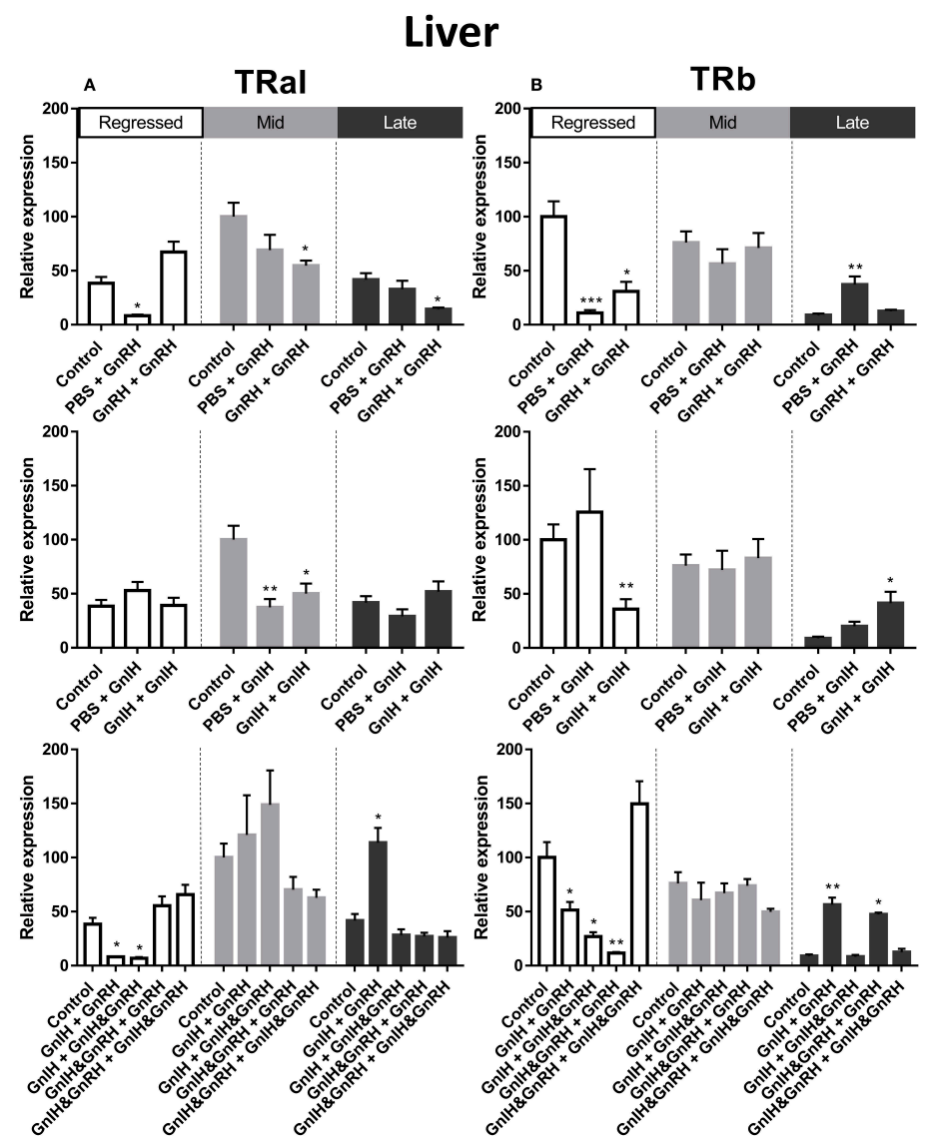
Effects of GnRH and GnIH on thyroid hormone receptors TRαI **(A)** andTRβ **(B)** mRNA levels in the liver of female goldfish at three seasonal reproductive stages (regressed, *n* = 4–9; mid recrudescence, *n* = 4–9; and late recrudescence *n* = 4–9). Treatments are denoted as 0 + 12 h injection. Levels of mRNA expression were detected using QPCR and normalized relative to β-actin. Results presented (mean ± SEM) are expressed as a percentage of the averaged relative expression levels in control (PBS+PBS) group at mid recrudescence. Each seasonal control group was placed in front of each set of hormone treatments for visual comparison. Asterisks indicate significance differences from controls (Kruskal-Wallis test followed by Dunn's multiple comparisons test, *p < 0.05, **p < 0.01, ***p = 0.001).

Thyroid hormones are important factors in reproduction and growth [for review see: (55)]. In female liver, TRαI transcript levels were observed to be significantly higher during mid recrudescent than in livers of regressed and mid recrudescent fish (Figure 3A). No clear correlation was observed between changes in TRαI mRNA levels and LH or GH secretion following hormone treatments. Double injections of GnRH decreased TRαI mRNA levels during mid and late stages of gonadal recrudescence in liver (Figure 8A). Treatments of GnIH alone, applied either as a single or double injection, reduced TRαI mRNA levels during mid recrudescence (Figure 8A). In late stages of recrudescence, GnIH + GnRH treatment increased TRαI mRNA levels (Figure 8A). In regressed stage fish, single GnRH injection treatment lowered TRαI mRNA levels and combination treatment with GnIH did not affect GnRH-induced responses in GnIH + GnRH and GnIH + GnIH & GnRH treatment groups (Figure 8A).

The seasonal expression pattern for liver TRβ mRNA was observed to be different from that of TRαI, with significantly higher levels in the regressed and mid recrudescence stages than at late recrudescence (Figure 3B). Similarly to TRαI, TRβ mRNA levels did not clearly correspond to changes in serum LH or GH levels elicited by hormone treatments. Treatments with GnRH alone reduced TRβ mRNA levels in liver of females during regressed stage, but a single GnRH injection increased TRβ mRNA levels during late recrudescence (Figure 8B). Double injections of GnIH reduced TRβ mRNA level in the liver in regressed gonadal stage females, but increased these transcript levels in late recrudescence (Figure 8B). In late recrudescence, treatments with GnIH + GnRH or GnIH & GnRH + GnRH increased TRβ mRNA levels (Figure 8B). All combination treatment groups in regressed gonadal stage fish reduced TRβ mRNA levels in liver, except treatment with GnIH & GnRH + GnIH & GnRH which had no effect on TRβ mRNA levels (Figure 8B). No significant changes were observed on TRβ transcript levels in mid stages of recrudescence with any treatment (Figure 8B).

#### Aromatase

**Figure 9:**
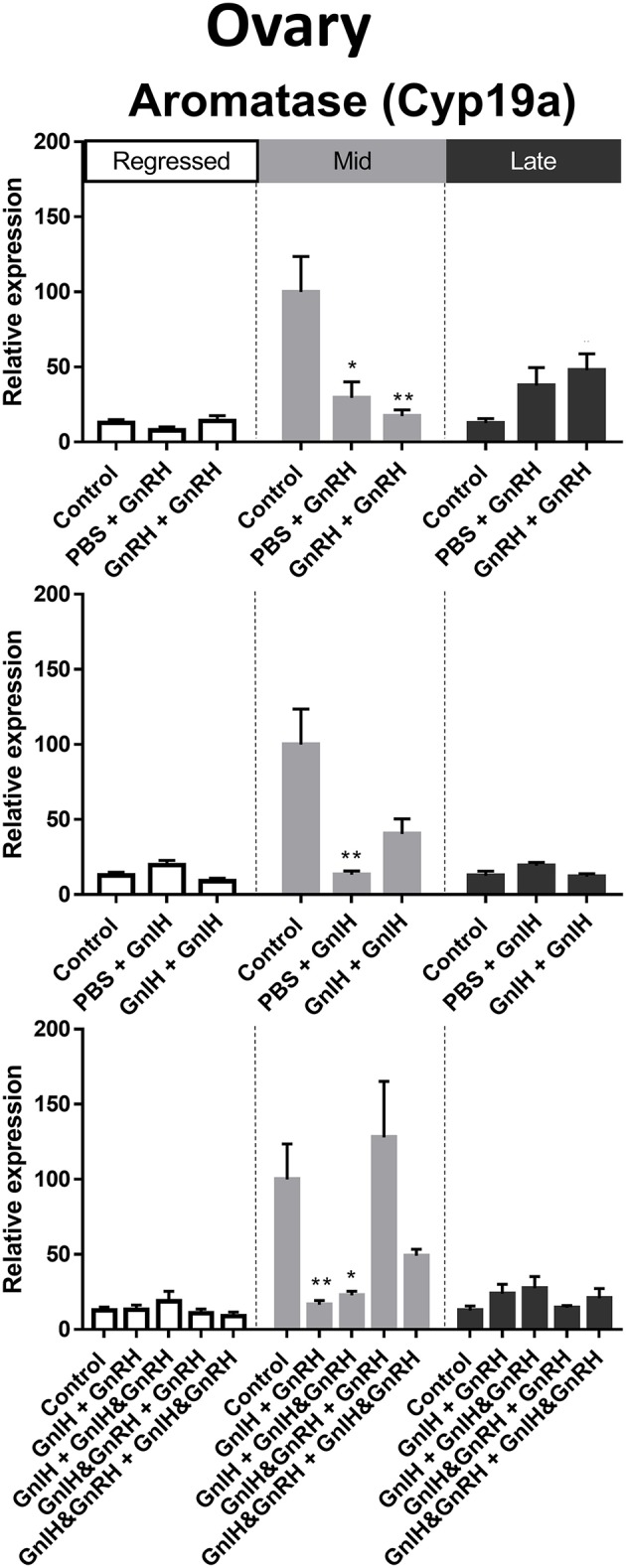
Effects of GnRH and/or GnIH on gonadal aromatase (Cyp19a) mRNA levels in the ovary of female goldfish at three seasonal reproductive stages (regressed, *n* = 4–9; mid recrudescence, *n* = 4–9; and late recrudescence *n* = 4–9). Treatments are denoted as 0 + 12 h injection. Levels of mRNA expression were detected using QPCR and normalized relative to GAPDH. Results presented (mean ± SEM) are expressed as a percentage of the averaged relative expression levels in control (PBS + PBS) group at mid recrudescence. Each seasonal control group was placed in front of each set of hormone treatments for visual comparison. Asterisks indicate significance differences from controls (Kruskal-Wallis test followed by Dunn's multiple comparisons test, *p < 0.05, **p < 0.01).

Gonadal aromatase (Cyp19a) is crucial for the aromatization of testosterones into estrogens (70). In the ovaries, relatively higher aromatase mRNA levels were observed in mid recrudescence compared to regressed and late recrudescence stages (Figure 3J). Partial correlation was observed between aromatase transcript expression and circulating LH concentrations following hormone treatments at late recrudescence stage (Figure 9). Treatments with either GnRH or GnIH alone reduced aromatase mRNA levels in ovaries during mid recrudescence and were without effect in regressed stage (Figure 9). Treatments with GnIH + GnRH or GnIH + GnIH & GnRH also lowered levels of aromatase mRNA in mid recrudescent ovaries, but not in regressed stage (Figure 9). At late stages of gonadal recrudescence, double GnRH injection treatment increase aromatase mRNA levels but no other treatments had any effects (Figure 9).

#### FSHR

**Figure 10:**
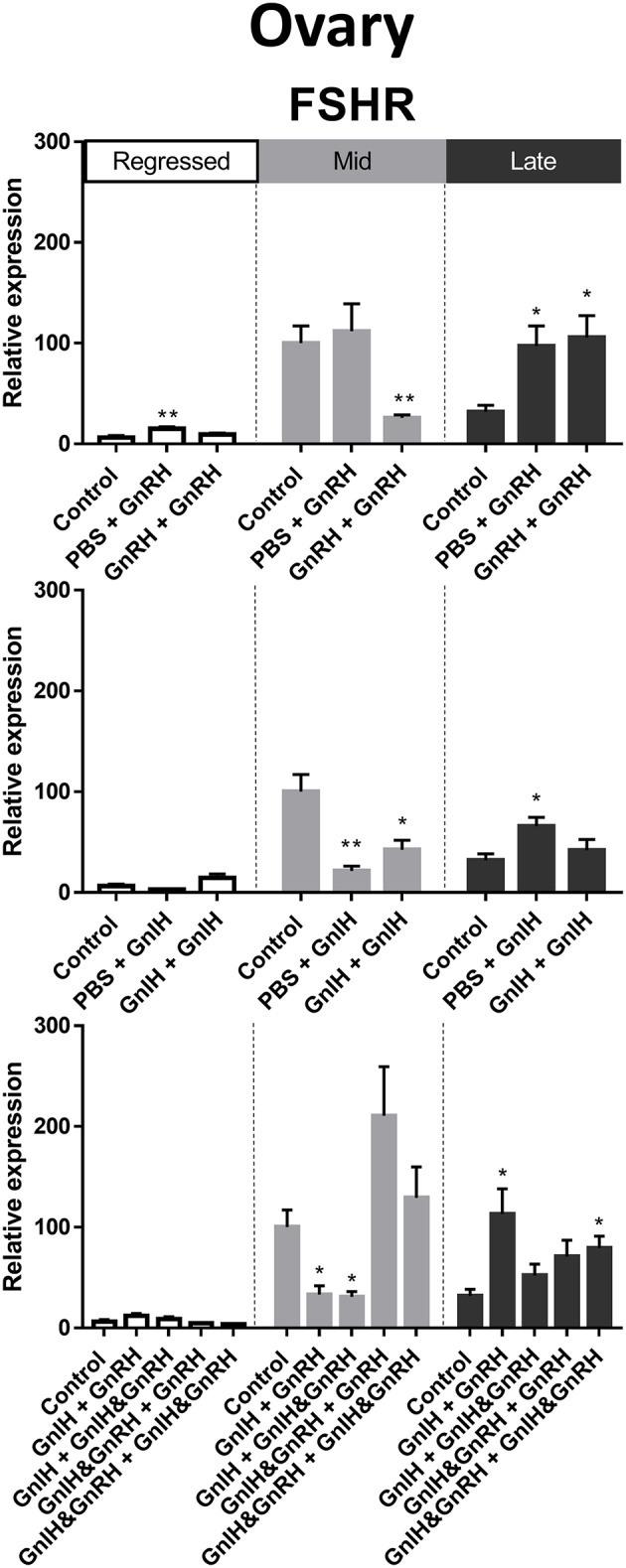
Effects of GnRH and GnIH on follicle stimulating hormone receptor (FSHR) mRNA levels in the ovary of female goldfish at three seasonal reproductive stages (regressed, *n* = 4–9; mid recrudescence, *n* = 4–9; and late recrudescence *n* = 4–9). Treatments are denoted as 0 + 12 h injection. Levels of mRNA expression were detected using QPCR and normalized relative to GAPDH. Results presented (mean ± SEM) are expressed as a percentage of the averaged relative expression levels in control group at mid recrudescence. Each seasonal control group was placed in front of each set of hormone treatments for visual comparison. Asterisks indicate significance differences from controls (Kruskal-Wallis test followed by Dunn's multiple comparisons test, *p < 0.05, **p < 0.01).

FSHR is a crucial facilitator of FSH actions on oocyte maturation and steroidogenesis (70). FSHR mRNA levels in ovary were highest during mid stages of recrudescence compared to regressed and late gonadal stages (Figure 3I). We observed partial correlation between FSHR mRNA levels and serum LH levels following GnRH treatments during late recrudescence phase. Treatments with either double or single injection of GnRH alone increased FSHR mRNA levels in ovaries of fish in late stages of recrudescence (Figure 10). In mid recrudescence, double injections of GnRH decreased ovarian FSHR mRNA levels, whereas treatment with a single injection of GnRH increased FSHR mRNA levels in regressed ovaries (Figure 10). Treatments with GnIH reduced FSHR mRNA levels in the ovary of mid recrudescent fish, while single GnIH injection treatments exerted a stimulatory influence at late recrudescence (Figure 10). Increased FSHR mRNA level was observed following combination GnIH + GnRH and GnIH & GnRH + GnIH & GnRH treatments in late recrudescence, but decreased transcript level was observed following GnIH + GnRH and GnIH + GnIH & GnRH treatments in mid recrudescence (Figure 10). Ovarian expression of the other gonadotropin receptor, LH receptor, was not quantified because of primer problems.

## Discussion

The present study provided insight into the neuroendocrine control of seasonal reproduction and growth in female goldfish. A seasonal pattern of change in basal serum LH concentrations in female goldfish was observed. The observed maximum circulating LH concentrations at late stages of recrudescence occurred in fish containing fully matured oocytes, and period of lower growth rates. This is consistent with a previous study in goldfish demonstrating maximum gonadosomatic index (GSI) in females during spring, lowest GSI during regressed phase, and increasing levels during mid recrudescence (71). In the present study, smaller ovary and follicle size was observed at regressed stage, increasing at mid recrudescence, and reaching a maximum size at late recrudescence, which also had higher circulating levels of LH. Marchant and Peter found higher rates of somatic and linear growth corresponding to the lowest GSI in female goldfish (71). In the present study, differences in basal circulating GH concentration in different reproductive seasons were not statistically significantly. In male fish, however, highest circulating levels of GH was observed at the regressed stage, compared to those at mid and late recrudescence (72). It should be noted that GH, as well as LH, contribute to stimulation of Vtg production, and thus ovarian maturation, in goldfish (73).

Treatment with GnRH had a stimulatory effect on serum LH levels at mid and late recrudescence stage, while GnRH-induced GH response was observed at regressed and late stages of recrudescence (summarized in Table S2). Previous studies demonstrated seasonally dependent changes and increased GnRH-induced LH release during late stages of gonadal recrudescence (36). GnRH has also been shown to increase LHβ subunit mRNA levels (2, 13). In the present study, serum GH concentrations were increased by both GnRH and GnIH injections at the regressed phase. In late recrudescence, however, only GnRH was stimulatory on circulating GH levels in female goldfish. Our results are consistent with a previous study demonstrating increases in serum GH levels and stimulation of GH mRNA expression during early and late gonadal recrudescence by both sGnRH and cGnRHII (14). A number of other studies also demonstrated GnRH stimulation of GH production in goldfish (11, 12, 15, 16, 21, 74), and other teleost species (20–22, 75). Thus, GnRH is an important factor in regulating GH and LH production and release in fish. The time course for both GnRH and GnIH were chosen based on previous studies (2, 13, 14, 35, 36, 63, 76). At 12 and 24 h following injection, GnRH significantly effects both GH and LH production as shown previously (2). GnRH-induced change in gonadotropins, in turn, effects production of gonadal steroids which can have feedback effect on the brain and pituitary within the time frames used in the present study (64, 65). Therefore, 12 and 24 h timepoints were chosen based on previous studies. It is possible that more information may be obtained by investigating effects of hormones at earlier time points and it may be a limitation of this study. However, adding more time points would limit our ability to investigate multifactorial control of pituitary function, using a factorial design.

To investigate the seasonal differences and interactions between GnRH and GnIH, we combined the two hormones following a factorial design. In goldfish, GnIH has been shown to both stimulate and inhibit LH release and synthesis in the pituitary, depending on the stages of gonadal maturation (35, 36). Various combinations of GnRH and GnIH in cell perifusion experiments resulted in different effects on GH and LH release (14, 36). The present results demonstrate stimulation of basal GH release by GnIH during the somatic growth phase of female goldfish, and GnIH inhibition of GnRH-stimulated GH secretion during late gonadal recrudescence but not in sexually regressed stage (summarized in Tables S3, S4). These findings are consistent with a previous study demonstrating direct action of GnIH on GH release *in vitro* (14). GnIH stimulation of GH release and production has been demonstrated in a variety of other vertebrate species including the grass puffer (*Takifugu alboplumbeus*) (38), cichlid fish (*Cichlasoma dimerus*) (34), bull frogs (*Rana catesbeiana*) (77), and rats (40). In the present study, treatment with GnIH resulted in inhibition of GnRH-induced LH secretion at late but not at mid gonadal recrudescence (summarized in Tables S3, S4). This is consistent with previous findings in goldfish both *in vivo* and *in vitro* (36). GnIH inhibition of gonadotropin gene expression has also been shown in cinnamon clownfish (*Amphiprion melanopus*) (33). In common carp (*Cyprinus carpio* L.), GnIH treatment reduced FSH and LH subunits expression, and minimum GnIH transcript levels were observed in the hypothalamus during spawning season (78). In the cichlid fish (*Cichlasoma dimerus*), treatment with cd-LPQRF-1 inhibited FSH and LH release, while cd-LPQRF-2 stimulated FSH release in cultured pituitary cells (34). In the flatfish, treatment with ssGnIH-2 did not affect LHβ expression but ssGnIH-3 lowered LHβ transcript levels (79). These studies provide evidence of species-specific changes in GnIH response depending on reproductive season. The general response elicited by GnIH in the female goldfish is largely inhibitory on gonadotropes and stimulatory on somatotropes in a seasonally related manner. Thus, both GnRH and GnIH are contributing factors in multifactorial regulation of seasonal growth and reproduction in female goldfish. Further studies investigating pituitary receptors of GnRH and GnIH could explain the seasonal changes in GH and LH observed in this study. Basal GnIH receptor mRNA levels in goldfish pituitary did not show significant changes during any seasonal gonadal stage (35). However, treatments of exogenous GnIH and GnRH affected GnIH receptor transcripts in mid and late recrudescence goldfish (36). It is possible that fluctuations in GnIH and GnRH receptor ratio may have important influence on seasonal regulation of somatotropes and gonadotropes in goldfish.

Thyroid hormones are also important in the control of reproduction in fish and other vertebrates (52, 53, 55, 56, 80). In goldfish, serum concentrations of T3 are high during summer when fish have maximum growth rates; circulating T3 then follows a decreasing trend from fall through spring, reaching minimum levels in the spawning period (60). In the present study, treatment with T3 was inhibitory on basal GH levels during mid recrudescence as well as reducing both GnRH- and/or GnIH-elicited GH responses during sexually regressed and late recrudescence stages. At mid and late recrudescence, T3 injection reduced GnRH-induced serum LH increases. However, co-injection with GnIH reduced inhibitory T3 effects on GnRH-induced LH response in mid recrudescence (summarized in Tables S2–S4). Previous results have demonstrated that T3 treatment, *in vitro*, reduced LH subunit mRNA levels during early recrudescence in mixed sex fish without affecting GH or gonadotropin subunits transcript levels at other stages of gonadal recrudescence in goldfish (51). Furthermore, injections with T3 at mid recrudescence stage were shown to have no effects on gonadotropin mRNA levels in female goldfish, but reduced LH subunit mRNA levels in male goldfish after 36 h (54). In addition, injections with T3 were shown to reduce ERα and Cyp19a transcript levels in the ovary of mid recrudescent fish (54). Taken together, T3 exerts inhibitory effects on HPG axis, and the control of pituitary somatotrope activities with seasonal variations. Thus, these results support the hypothesis that T3 is an important contributor to seasonal control of GH and LH in female goldfish. Furthermore, in view of the previously described progressive decrease in circulating T3 levels as gonadal recrudescence advances, the reduction of the negative T3 influence on the HPG axis, as well as on pituitary GH release, is likely an important neuroendocrine event for the seasonal switch from somatic growth to gonadal maturation.

It is important to note that goldfish cannot be sexed accurately based on secondary sex characteristics, especially during the regressed gonadal stage. During the experiment, fish were housed in tanks that contained mature males and females that were randomly distributed. At the time of dissection, it was possible to accurately sex the fish. Due to this potential limitation, there were variations among the replicate numbers for different treatment groups. However, the majority of groups contained large replicate numbers as stated for each figure. Another possible limitation of this study was variation in size of fish in different seasons. All fish were post-pubertal and sexually mature and expected to have similar hormonal profiles and responsiveness to same treatments regardless of variations in size. However, we cannot ignore the influence that size may have and consider variations in size of the fish as a limitation of this study.

The present study investigated tissue- and season-specific transcript levels of several genes related to growth and reproduction in the liver and ovary. The results show similar seasonal pattern for ERα and ERβI mRNA levels in the female liver and ovaries with the highest level of these transcripts at mid recrudescence in both tissues. A similar seasonal pattern of ERα and ERβI transcript levels were also observed in male goldfish liver, although peak levels of ERα and ERβI mRNA in testes where different during late recrudescence (72).

Both ERα and ERβI are involved in stimulating vitellogenesis in female fish (80–82). Vtg production starts during early recrudescence and continues through mid-recrudescence until oocytes are fully mature in late recrudescence. This cycle is clearly seen in female liver ERα and ERβI transcript levels and correlates with higher Vtg mRNA levels in mid and late recrudescence. Interestingly, intermediate levels of ERα and ERβI transcript levels were seen in the regressed and late recrudescence states in the liver and ovary, respectively. The intermediate levels of ER expression in the liver during earlier stage of gonadal recrudescence could be the result of T3 priming effect for vitellogenesis (61). The Vtg gene is also found in males but is not usually expressed at significant levels due to lack of sufficient circulating levels of estrogens. Never the less, Vtg level was found to follow a similar seasonal pattern as female liver, although, the levels of ERα and Vtg mRNA in male liver are much lower compared females (72).

The present results also revealed higher levels of ERα and ERβI transcript levels in ovaries at the mid recrudescence stage which corresponds with higher expression in ovarian aromatase and FSHR transcript levels. In goldfish and other teleosts, FSH also has steroidogenic activity (83, 84) and thus the changes in FSHR expression at mid recrudescence is not solely related to gametogenesis, but is also relevant for the increase in total steroidogenic capacity at this reproductive stage. ERs and estrogen synthesis are crucial components of oocyte maturation and female reproduction (85, 86). FSH effects on increasing follicular cell activities and cell proliferation are largely mediated through estrogens and ERs, and E2 positive feedback on follicular cell proliferation further increases steroidogenic capacity of the maturing follicles (85). The increasing FSH and E2 levels during early recrudescence triggers liver vitellogenesis, and as the oocyte reaches final maturation, FSH and E2 levels decrease (85). E2 and GPR30 (a membrane estrogen receptor) has been demonstrated to inhibit spontaneous oocyte maturation in fish (86). In goldfish gonads, estradiol stimulates ERs in a time and dose dependant manner (81). Estrogens and ERs have been shown to be involved in testicular development (87, 88) and undergo seasonal variations together with ERβI, FSHR, and aromatase mRNA which are produced at higher levels during late recrudescence stage compared to other seasons (72). It should be noted that testicular aromatase transcript levels are generally lower than ovarian aromatase transcript levels (72). The observed higher mRNA levels of ERα, ERβI, and aromatase in the ovary compared to testis is likely the result of the roles these factors play in the regulation of ovarian function and development. Transcript level for GH, LH subunits, LHR and a number of other hormones and receptors were not investigated in the present study. There is already information on GHR mRNA levels in goldfish at mid-late and regressed gonadal stages (73). Circulating levels of E2 have also been extensively measured throughout the seasonal cycles of goldfish (51, 60, 89, 90). We did not study these factors in the present study, but recognize that quantifying these receptors, and serum E2 levels in response to treatments would have helped to better understand and link the hormonal control of reproduction and growth between pituitary, ovary, and liver.

Results in the present study demonstrated seasonal fluctuations in TRαI, TRβ, and IGF-I transcript levels, which were similar to those found in male goldfish liver (72). This is consistent with previous observations in goldfish (91). TRαI transcript levels in female liver were found to be higher in mid recrudescence, which correlated with higher IGF-I transcript levels. TRβ transcript level was observed to be higher in regressed and mid recrudescence stages compared to late recrudescence. It was shown previously that thyroid hormones play a role in stimulating the vitellogenic capacity of liver by elevating ERα expression and through actions on both TRα and TRβ (61). Higher TRβ expression during earlier gonadal recrudescence stages possibly contributes to T3 priming effect in the liver of female goldfish discussed earlier (61).

We further examined transcript levels of the same genes in response to treatments with GnRH and GnIH in the liver and ovaries of goldfish. However, the majority of the results do not demonstrate clear correlation with changes in LH and GH serum levels. Notably, although changes in ovarian ERs, FSHR and aromatase transcript levels in GnRH- and/or GnIH-treated fish were correlated with one another at mid recrudescence and reflected the close relationships between these elements in the neuroendocrine regulation of ovarian steroidogenesis and development at this reproductive stage (84), these changes mirrored neither serum LH and GH concentrations in the treatment groups. In addition at mid recrudescence, a time when ovarian steroidogenesis and yolk incorporation should be increasing (89), GnRH paradoxically exerted inhibitory effects on the levels of these ovarian gene transcripts, and liver Vtg mRNA levels were reduced by single injection of GnRH both in the absence and in the presence of GnIH. It is possible that direct actions of GnRH and GnIH on peripheral tissues, including liver and gonads, as well as changes in pituitary LH and GH release may have contributed to these complex results. Teleost fish lack a hypothalamo-hypophyseal portal system, and GnIH and GnRH directly act on the pituitary cells by innervations (92). Circulating levels of GnRH or GnIH are undetectable or lower than 1 pg/ml and therefore unlikely to elicit peripheral effects via endocrine actions (93, 94). However, with injections, we introduced higher levels of GnIH and GnRH in the circulation and it would be possible for these peptides to exert direct actions on the liver and ovaries. In fish and other vertebrates, the expression of GnRH and GnIH and their receptors, as well as their direct action have been demonstrated in peripheral tissues including liver and gonads (95–104). For example, although *in vitro* GnRH treatments of goldfish ovarian follicles in culture stimulated germinal vesicle breakdown, GnRH also attenuated gonadotropin- and progesterone-induced oocyte meiosis and steroidogenesis (105–107). GnIH direct inhibition of steroidogenesis in follicles could also contribute to changes in transcript level of various genes (108, 109). On the other hand, partial correlations were observed between ovarian ERα, ERβI, and FSHR transcript levels and GnRH-stimulated LH responses during late recrudescence in the present study. Consistent with the known ability of GH to directly stimulate IGF-I production in hepatocytes (110), increased GH serum concentrations induced by GnRH in late recrudescence was correlated with increased IGF-I mRNA levels in the liver. Thus, the observed responses in the different transcripts in liver and ovary to hormone treatments could be the consequence of changes in circulating GH and LH levels in addition to direct actions on the liver and ovary.

Dopamine is another important factor that contributes to the seasonal shift between growth and reproduction because it is known to reciprocally stimulate somatotropes and inhibit gonadotropes in goldfish (66, 111–117). Maximum somatotrope responsiveness to dopamine was demonstrated during growth seasons and lowest response was seen in sexually mature fish (114). Dopamine is possibly a key factor in reciprocal regulation of basal and GnRH and GnIH regulation of gonadotropes and somatotropes. Whether and how dopamine and other neuroendocrine factors known to exert direct reciprocal influences on gonadotropes and somatotropes, such as norepinephrine (118–120) and serotonin (121–123), co-ordinate and interact with GnRH, GnIH, and thyroid hormones in the integrated neuroendocrine control of growth and reproduction would be important areas for future studies.

## Conclusion

In summary, the present study demonstrates that GnRH and GnIH are factors in the regulation of LH and GH levels. GnIH inhibits GnRH-induced LH and GH release in fish at late stage of recrudescence. T3 exerts mainly inhibitory action on basal and GnRH- and/or GnIH-induced GH release. T3 inhibitory action on GnRH-induced LH release is reduced in the presence of GnIH in a gonadal state-dependent manner. Overall regulation of growth and reproduction is multifactorial and involve GnRH, GnIH, and T3. These results help to better understand the reciprocal regulation of seasonal reproduction and growth in female goldfish and other seasonally reproducing animals.

## Data Availability Statement

All datasets generated for this study are included in the article/Supplementary Material.

## Ethics Statement

The animal study was reviewed and approved by University of Calgary Ethics Committee for use and care of experimental animals.

## Author Contributions

YM, CL, and HH designed research. Research performed by YM and CL. YM analyzed data. CL, JC, and HH provided intellectual input on the data analysis. YM, JC, and HH wrote the paper. JC provided funding and oversight on radioimmunoassays. HH provided funding, intellectual input, and oversight on experimental design and data analysis.

### Conflict of Interest

The authors declare that the research was conducted in the absence of any commercial or financial relationships that could be construed as a potential conflict of interest.
