# Supplementary material for: Seasonal Related Multifactorial Control of Pituitary Gonadotropin and Growth Hormone in Female Goldfish: Influences of Neuropeptides and Thyroid Hormone
Source: Front Endocrinol (Lausanne). 2020 Apr 7;11:175. doi: 10.3389/fendo.2020.00175 (PMC7154077; doi:10.3389/fendo.2020.00175)
Supplement: Supplementary file 1 [file Table_1.DOCX]

**Supplementary Tables and Figures**

Table S.1. Additional primer information for all primers used in QPCR analysis. Efficiency of all primers were within 90-100%.

| Gene | Annealing Temperature (°C) | Tm (°C) | Amplicon Length (bp) |
| --- | --- | --- | --- |
| β-actin  (Liver) | 57 | 81.5 | 94 |
| GAPDH  (Testes) | 57 | 84.5 | 152 |
| Vtg | 55 | 82 | 168 |
| IGF-I | 57.1 | 88 | 154 |
| ERα | 55 | 85 | 116 |
| ERβI | 55 | 82.5 | 144 |
| TRαI | 55 | 84 | 95 |
| TRβ | 55 | 86.5 | 108 |
| FSHR | 56 | 83.5 | 153 |
| Cyp19a1  (aromatase) | 58 | 81.5 | 129 |

Table S.2. Effects of sGnRH on pituitary GH and LH release during regressed phase, mid recrudescence, and late recrudescence on female goldfish. RIAs were used to detect for LH and GH serum levels in experimental groups with and without addition of T3 at each injection. In the case of control group (0 hours: PBS, 12 hours: PBS) with T3, only T3 was administered. Up arrow indicates increased levels from control, down arrows indicate lower levels from control, and bar indicates no differences from control.

|  | Reproduction | | | | | | | | |
| --- | --- | --- | --- | --- | --- | --- | --- | --- | --- |
|  | Regressed | | | Mid Recrudescence | | | Late Recrudescence | | |
| 0hrs  12hrs | PBS  PBS | PBS  GnRH | GnRH  GnRH | PBS  PBS | PBS  GnRH | GnRH  GnRH | PBS  PBS | PBS  GnRH | GnRH  GnRH |
| LH: without T3 |  |  |  |  |  |  |  |  |  |
| LH: with T3 |  |  |  |  |  |  |  |  |  |
|  | Growth | | | | | | | | |
| GH: without T3 |  |  |  |  |  |  |  |  |  |
| GH: with T3 |  |  |  |  |  |  |  |  |  |

Table S.3. Effects of gfGnIH on pituitary GH and LH release during regressed phase, mid recrudescence, and late recrudescence on female goldfish. RIAs were used to detect for LH and GH serum levels in experimental groups with and without addition of T3 at each injection. In the case of control group (0 hours: PBS, 12 hours: PBS) with T3, only T3 was administered. Up arrow indicates increased levels from control, down arrows indicate lower levels from control, and bar indicates no differences from control.

|  | Reproduction | | | | | | | | |
| --- | --- | --- | --- | --- | --- | --- | --- | --- | --- |
|  | Regressed | | | Mid Recrudescence | | | Late Recrudescence | | |
| 0hrs  12hrs | PBS  PBS | PBS  GnIH | GnIH  GnIH | PBS  PBS | PBS  GnIH | GnIH  GnIH | PBS  PBS | PBS  GnIH | GnIH  GnIH |
| LH: without T3 |  |  |  |  |  |  |  |  |  |
| LH: with T3 |  |  |  |  |  |  |  |  |  |
|  | Growth | | | | | | | | |
| GH: without T3 |  |  |  |  |  |  |  |  |  |
| GH: with T3 |  |  |  |  |  |  |  |  |  |

Table S.4. Effects of sGnRH and gfGnIH on pituitary GH and LH release during regressed phase, mid recrudescence, and late recrudescence on female goldfish. RIAs were used to detect for LH and GH serum levels in experimental groups with and without addition of T3 at each injection. In the case of control group (0 hours: PBS, 12 hours: PBS) with T3, only T3 was administered. Up arrow indicates increased levels from control, down arrows indicate lower levels from control, and bar indicates no differences from control.

| `Reproduction | | | | | | | | | | | | | | | |
| --- | --- | --- | --- | --- | --- | --- | --- | --- | --- | --- | --- | --- | --- | --- | --- |
|  | Regressed | | | | | Mid Recrudescence | | | | | Late Recrudescence | | | | |
| 0hrs | PBS | GnIH | GnIH | GnIH GnRH | GnIH GnRH | PBS | GnIH | GnIH | GnIH GnRH | GnIH GnRH | PBS | GnIH | GnIH | GnIH GnRH | GnIH GnRH |
| 12hrs | PBS | GnRH | GnIH GnRH | GnRH | GnIH GnRH | PBS | GnRH | GnIH GnRH | GnRH | GnIH GnRH | PBS | GnRH | GnIH GnRH | GnRH | GnIH GnRH |
| LH: without T3 |  |  |  |  |  |  |  |  |  |  |  |  |  |  |  |
| LH: with T3 |  |  |  |  |  |  |  |  |  |  |  |  |  |  |  |
| Growth | | | | | | | | | | | | | | | |
| GH: without T3 |  |  |  |  |  |  |  |  |  |  |  |  |  |  |  |
| GH: with T3 |  |  |  |  |  |  |  |  |  |  |  |  |  |  |  |
